# High Inter-Rater Reliability of Manual Segmentation and Volume-Based Tractography in Healthy and Dystrophic Human Calf Muscle

**DOI:** 10.3390/diagnostics11091521

**Published:** 2021-08-24

**Authors:** Johannes Forsting, Marlena Rohm, Martijn Froeling, Anne-Katrin Güttsches, Matthias Vorgerd, Lara Schlaffke, Robert Rehmann

**Affiliations:** 1 Department of Neurology, BG-University Hospital Bergmannsheil, Ruhr-University Bochum, 44789 Bochum, Germany; Marlena.Rohm@rub.de (M.R.); Anne.Guettsches@rub.de (A.-K.G.); Matthias.Vorgerd@rub.de (M.V.); Lara.Schlaffke@rub.de (L.S.); Robert.Rehmann@rub.de (R.R.); 2 Heimer Institute for Muscle Research, BG-University Hospital Bergmannsheil, 44789 Bochum, Germany; 3 Department of Radiology, University Medical Centre Utrecht, 3584 Utrecht, The Netherlands; M.Froeling@umcutrecht.nl; 4 Department of Neurology, Klinikum Dortmund, University Witten-Herdecke, 44137 Dortmund, Germany

**Keywords:** diffusion tensor imaging, muscle MRI, tractography, quantitative MRI, calf musculature, neuromuscular diseases

## Abstract

Background: Muscle diffusion tensor imaging (mDTI) is a promising surrogate biomarker in the evaluation of muscular injuries and neuromuscular diseases. Since mDTI metrics are known to vary between different muscles, separation of different muscles is essential to achieve muscle-specific diffusion parameters. The commonly used technique to assess DTI metrics is parameter maps based on manual segmentation (MSB). Other techniques comprise tract-based approaches, which can be performed in a previously defined volume. This so-called volume-based tractography (VBT) may offer a more robust assessment of diffusion metrics and additional information about muscle architecture through tract properties. The purpose of this study was to assess DTI metrics of human calf muscles calculated with two segmentation techniques—MSB and VBT—regarding their inter-rater reliability in healthy and dystrophic calf muscles. Methods: 20 healthy controls and 18 individuals with different neuromuscular diseases underwent an MRI examination in a 3T scanner using a 16-channel Torso XL coil. DTI metrics were assessed in seven calf muscles using MSB and VBT. Coefficients of variation (CV) were calculated for both techniques. MSB and VBT were performed by two independent raters to assess inter-rater reliability by ICC analysis and Bland-Altman plots. Next to analysis of DTI metrics, the same assessments were also performed for tract properties extracted with VBT. Results: For both techniques, low CV were found for healthy controls (≤13%) and neuromuscular diseases (≤17%). Significant differences between methods were found for all diffusion metrics except for λ_1_. High inter-rater reliability was found for both MSB and VBT (ICC ≥ 0.972). Assessment of tract properties revealed high inter-rater reliability (ICC ≥ 0.974). Conclusions: Both segmentation techniques can be used in the evaluation of DTI metrics in healthy controls and different NMD with low rater dependency and high precision but differ significantly from each other. Our findings underline that the same segmentation protocol must be used to ensure comparability of mDTI data.

## 1. Introduction

To monitor and identify neuromuscular diseases (NMD), quantitative MRI (qMRI) protocols are used as possible surrogate biomarkers [1]. A method with emerging importance is muscle diffusion tensor imaging (mDTI), which provides information about local water diffusion and muscle tissue microstructure by measuring water diffusion in high resolution [2]. Since mDTI metrics are known to vary between different muscles, separation of different muscles is essential to achieve muscle-specific diffusion parameters [3].

Traditional segmentation techniques include commonly used manual segmentation-based analysis (MSB) and tractography algorithms [4]. In MSB, individual muscles are delineated on every slice (e.g., of T1w-images), which results in a 3-dimensional muscle volume. By superimposing those muscle volumes on mDTI maps, the diffusion metrics of the voxels within these masks are extracted and analyzed.

Another approach is to perform whole muscle tractography in a previously defined muscle volume that is registered on a DTI dataset. Subsequently, the diffusion metrics are extracted by tract-based sampling [5]. This technique is called volume-based tractography (VBT). In a previous study by our group, VBTshowed high inter-rater reliability and high sensitivity to detect intermuscular variances regarding diffusion parameters in healthy thigh muscles [6]. An advantage of tractography is that it provides additional information about the muscle architecture and microstructure, such as fiber tract length, pennation angle, muscle volume, and fiber tract count [7]. Such tractography-based parameters can provide additional information about the tissue microstructure of a diseased muscle [8,9].

In this context, myopathic muscle degeneration can result in a high degree of fatty infiltration and an increase in connective tissue as well as inflammation which all can influence diffusion metrics. A high degree of fat infiltration can complicate muscle segmentation due to deviating anatomy [10]. Since automatic segmentation algorithms are still in evaluation, muscle segmentation is usually done manually [11,12]. As manual segmentation is time- and cost-consuming and NMD are rare diseases, the pooling of data plays an important role in the application in clinical studies [13]. Therefore, it is essential to know to what extent different raters influence diffusion metrics assessed by different segmentation techniques. Inter-rater reliability of VBT in comparison with gold standard MSB has been validated in healthy thigh muscles, but little is known about how they compare in NMD [6]. A low rater dependency would suggest that pooling data between multiple centers is feasible.

Keller et al. pooled data from patients with several different myopathies to test for the influence of fatty tissue in mDTI imaging with and without selective ROI placement [14]. They argued that fatty degeneration is the common terminal route of muscle degeneration in many myopathies, and data can be pooled to test for segmentation. Here, we aimed to pool muscle data from different myopathies to test the inter-rater reliability and quality of data resulting from MSB and VBT muscle segmentation.

The purpose of this study was to assess the inter-rater reliability of VBT in comparison with MSB in healthy and dystrophic calf muscles. In a second step, we intended to evaluate rater dependency of tract properties extracted from VBT in healthy and diseased muscles.

## 2. Materials and Methods

### 2.1. Study Population

Twenty healthy controls (10 females) and 18 patients (11 females) with different NMD (Myotonic Dystrophy: *n* = 8; Pompe disease: *n* = 2; Inclusion Body Myositis: *n* = 4; Limb-Girdle Muscular Dystrophy: *n* = 4) were included in this study. The mean age of the control group was 33 years (SD 6 years), while the mean age in the NMD group was 58 years (SD 14 years). Mean BMI was 23.1 ± 2.2 in the control group and 28.2 ± 5.1 in the NMD group. Inclusion criteria for healthy controls included no strength exercise of leg muscles 5 days prior to enrolment and no leg injuries 12 months prior to examination. The study protocol was approved by the local ethics committee (Ruhr University Bochum No 15-5281).

### 2.2. Data Acquisition

An MRI was performed using a 3T MRI system (Achieva 3T X, Philips) and a 16-channel torso XL coil. Participants were instructed to lie still in a feet-first supine position. The MRI protocol was similar to our previous study in thigh muscles and included proton density-weighted (PD), T2-weighted (T2w), diffusion-weighted imaging (DWI), and a Dixon fat-quantification sequence (mDixonquant) in an axial slice order from proximal to distal (total acquisition time 18 min) [6]. To avoid shimming artifacts due to the large field of view (FOV), the calf region was divided into two FOVs of 480 × 264 × 150 mm^3^ along the *z*-axis (stacks). For accurate merging, the stacks had an overlap of 10 mm.

Using a voxel size of 1.5 × 1.5 × 3.0 mm^3^ and a repetition time/echo time (TR/TE) of 1646/15 ms, a PD sequence with turbo spin-echo (TSE) readout was achieved. A T2w TSE sequence was acquired with fat suppression (SPAIR) using the following parameters: voxel size 1.5 × 1.5 × 3.0 mm^3^; TR/TE 11 422/53 ms. The following parameters were used to obtain a diffusion-weighted spin echo-echo planar imaging acquisition: voxel size 3.0 × 3.0 × 6.0 mm^3^; TR/TE 3819/46 ms; SPAIR fat suppression; SENSE: 2; 17 gradient directions with b = 400 s/mm^2^ and three non-diffusion-weighted images (b = 0 s/mm^3^). A separate noise measurement was performed by turning off the RF and imaging gradients. Finally, a Dixon fat-quantification sequence (mDixonquant) was acquired with the following parameters: voxel size 1.5 × 1.5 × 3.0 mm^3^; TR/TE 7.2/1.21; 2.21; 3.21; 4.21; 5.21; 6.21 ms.

### 2.3. Data Processing

Data were preprocessed similar to Schlaffke et al. using QMRITools software running under Mathematica 11 [15,16]. In short, DWI images were merged, denoised, and motion-corrected by registration to the T2 image. Affine registration was used and aligned with T2w data using non-rigid registration (1000 iterations, b-spline spacing 120, 80, 80), including the rotation of the b-matrix to correct for subject motion and eddy current distortions. Tensor calculation was performed using the MATLAB-based toolbox ExploreDTI applying iterative weighted least squares with outlier rejection (REKINDLE) [17,18]. The mDixonquant sequence reconstructs fat-fraction maps directly on the MR host computer.

### 2.4. Muscle Segmentation and Tractography

**Figure 1:**
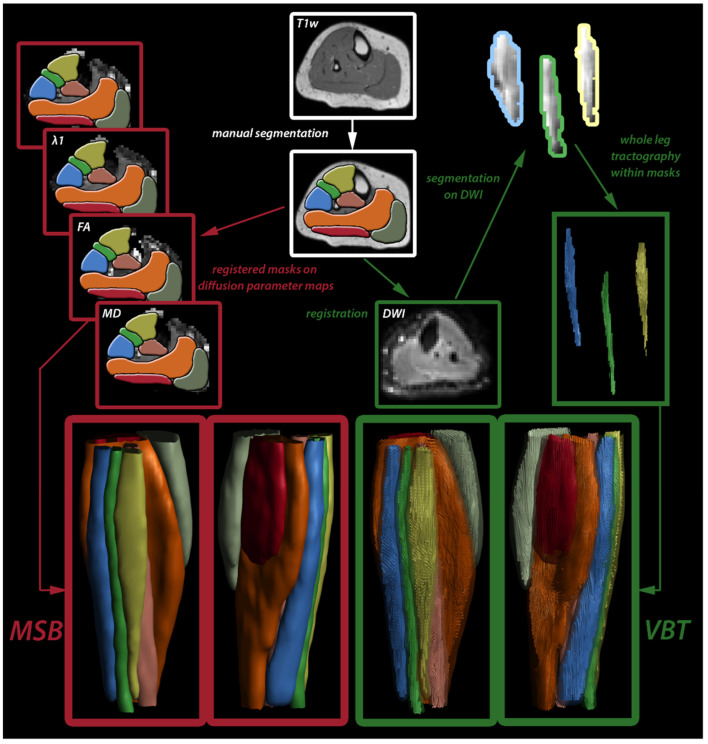
Overview of segmentation processes (MSB = manual segmentation, VBT = volume-based tractography).

Segmentation of seven calf muscles (extensor digitorum, gastrocnemius lateralis and medialis, peroneus group, soleus, tibialis anterior and posterior) was performed by two independent raters (for an overview, see Figure 1). Avoiding subcutaneous fat and fascia, these muscles were manually segmented on all slices of the PD image using a 3D slicer (3D-slicer 4.4.0, https://www.slicer.org, accessed on 21 August 2021). Adjacent muscles with high fatty infiltration were separated by considering anatomical features.

To obtain muscle-specific fat-fractions, the delineated masks were superimposed on mDixonquant fat-fraction maps. Whole calf fat-fraction data were calculated as the mean of the average fat-fraction of the individual muscles. SNR of diffusion images was calculated as the local average signal divided by the local noise sigma as described before [15,19].

Afterward, for MSB analysis, the resulting masks were smoothed and eroded by one voxel to avoid partial volume effects of non-muscular tissue and registered to the diffusion space to extract the diffusion metrics of FA, MD, λ_1,_ and radial diffusivity (RD)) for each muscle.

For VBT, the preprocessed diffusion data were masked based on the segmentation for each muscle. Only within these resulting segments of diffusion data, whole muscle tractography was performed with the toolbox MRIToolkit using the following fiber tracking stop parameters: maximum angle 15°, step size 1.5mm, FA range 0.1–0.6 [18,20]. The DTI parameters were extracted for each individual muscle using tract-based sampling (see Figure 1) [6]. Furthermore, tract properties tract density (TD)—defined as the number of tracts per volume—mean tract length (MTL), volume (Vol), and mean angle were calculated for resulting fiber tracts for each muscle separately.

### 2.5. Statistical Analysis

All statistical analyses were performed using SPSS V24 (IBM, Ehningen, Germany). Coefficients of variance (CV) were calculated as standard deviation/mean value. To determine differences in DTI-derived parameters (FA, MD, λ_1_–_3_, RD) between MSB and VBT, paired *t*-tests were performed. The significance level for all tests was set at *p* < 0.05. Correlation analysis (Pearson and intraclass correlation coefficient) and Bland-Altman plots were used to assess inter-rater reliability in healthy individuals. In a second step, these assessments were also performed in dystrophic muscles. Then correlation analysis was performed separately for muscles with fatty infiltration (FF > 10%) to evaluate the stability of rater influence in fatty infiltrated muscle. Correlation and Bland-Altman analysis were also completed to compare MSB and VBT directly. Finally, correlations and Bland-Altman plots were analyzed for tract properties extracted by VBT to assess inter-rater reliability.

## 3. Results

**Figure 2:**
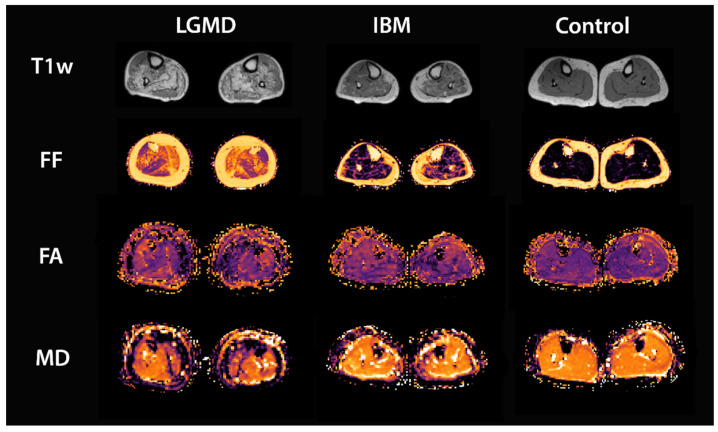
Example images of the applied MRI sequences: T1w, mDixon fat fraction (FF), FA and MD for lower legs of an LGMD, an IBM patient, and a healthy control.

**Figure 3:**
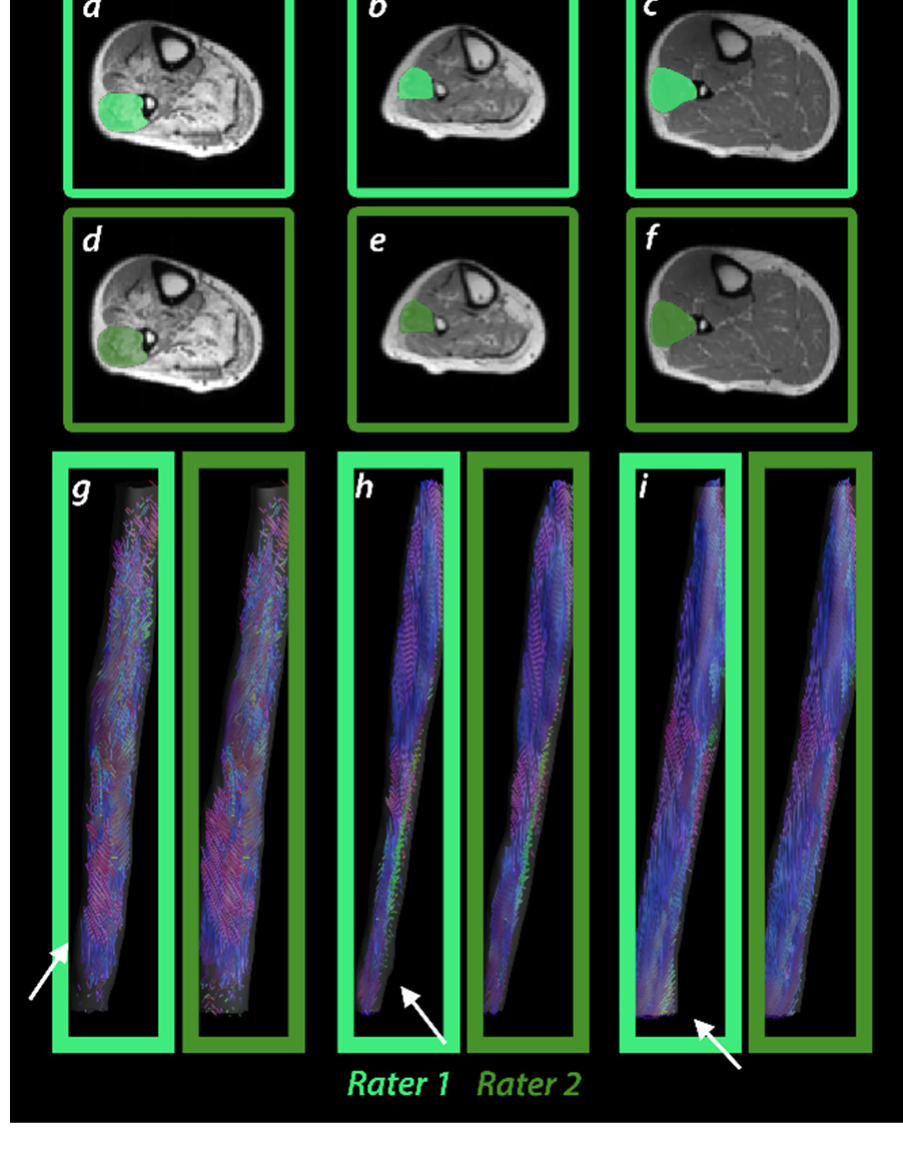
Segmentation of peroneal muscle group and tractography results for different raters in patients with LGMD (**a**,**d**,**g**), IBM (**b**,**e**,**h**), and a healthy control (**c**,**f**,**i**).

All scans were successfully performed in all participants. A representative T1w image, as well as a fat-fraction map, an FA map, and an MD map of an LGMD patient, an IBM patient, and a healthy control are shown in Figure 2. MSB and VBT were successfully used to segment seven different calf muscles (extensor digitorum, gastrocnemius medialis, gastrocnemius lateralis, peroneus muscle group, soleus, tibialis anterior, tibialis posterior) in all datasets. A segmentation of the peroneus muscle group in different individuals and fiber tracts obtained by VBT is visualized in Figure 3.

SNR of diffusion images was good for healthy controls (59.4 ± 16.9) and NMD group (58.1 ± 20.2) [19]. Fat fraction in NMD varied from 0.03 to 0.78 (mean: 0.16 ± 0.15). Mean FA values were 0.22 ± 0.03 for MSB and 0.21 ± 0.03 for VBT in both healthy control and NMD group. In contrast, mean MD values were lower in NMD group (MSB: 1.54 ± 0.18; VBT: 1.57 ± 0.16) compared with healthy individuals (MSB: 1.58 ± 0.10; VBT: 1.60 ± 0.12) for both segmentation techniques (see Table 1). To assess inter-subject variability, CV were calculated which were comparable between MSB and VBT for both study groups (healthy: MSB 0.06–0.13; VBT 0.07–0.12; NMD: MSB 0.11–0.17; VBT 0.10–0.17). Overall, CV were comparable between both methods in both groups. Paired *t*-tests revealed significant differences between the two segmentation methods for all diffusion metrics except from λ_1_ in the healthy control group and λ_1_ and λ_2_ in the NMD group.

ICC and Cronbach’s α could show an excellent agreement between both raters regarding mDTI values for both patients and controls and in both methods with slight advantages for MSB (see Table 2).

**Figure 4:**
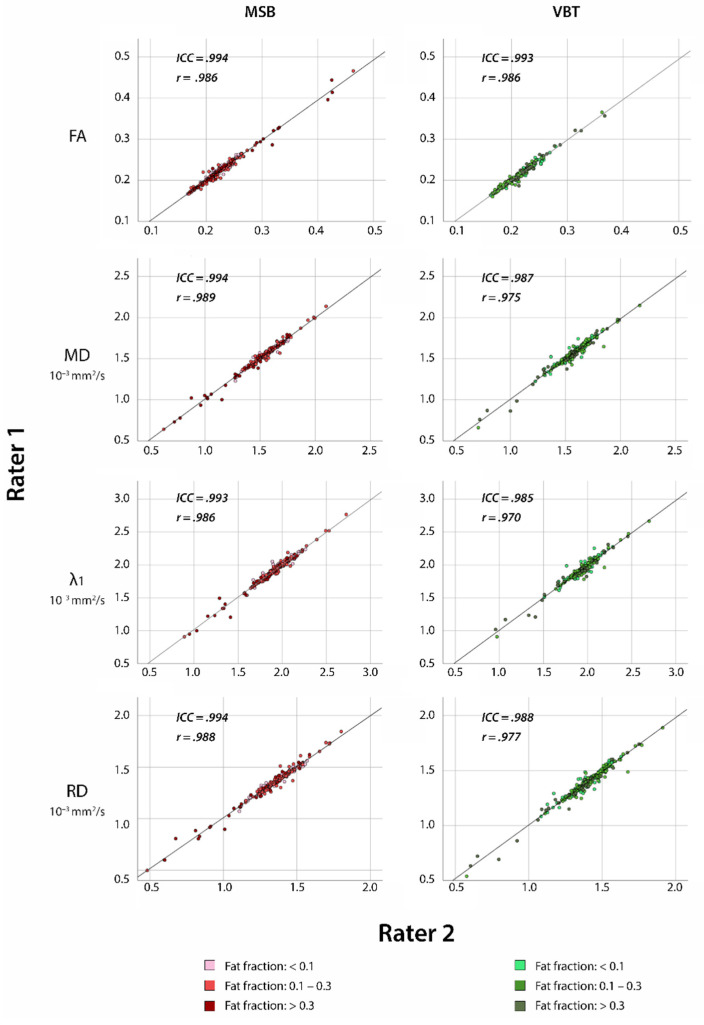
Scatter plots of correlation between inter-rater measurements for VBT and MSB in patients with neuromuscular diseases with Pearson correlation coefficient (r) and intraclass correlation coefficient (ICC) included in each graph.

**Figure 5:**
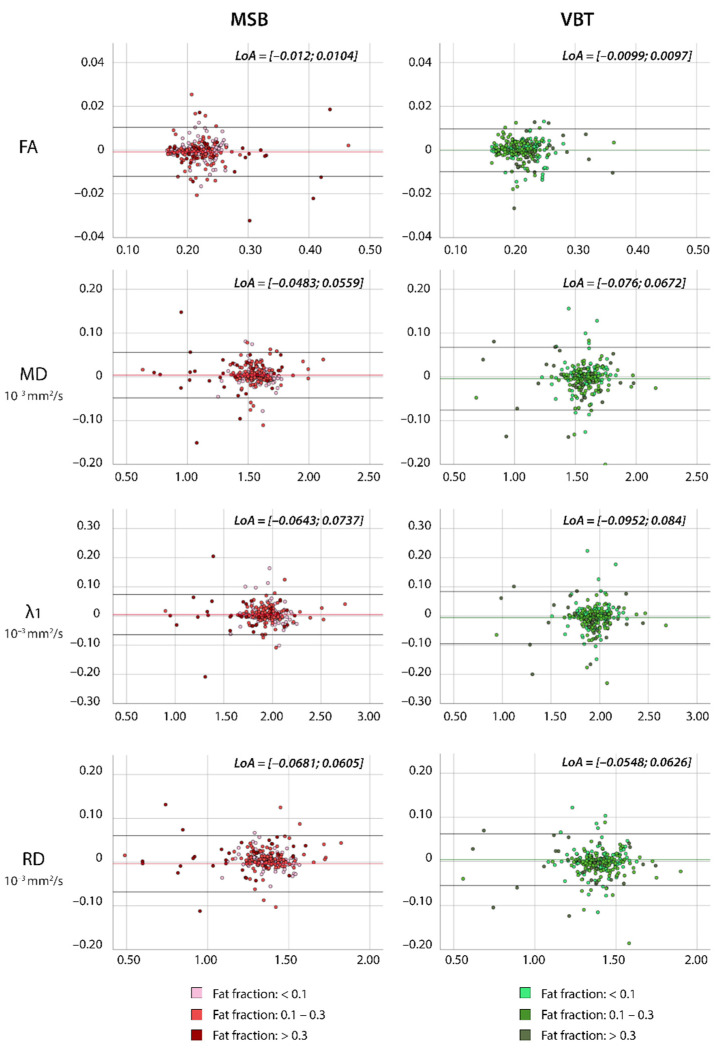
Bland-Altman plots of inter-rater measurements for MSB and VBT in individuals with neuromuscular diseases. The x-value shows the mean of two raters, and the y-value the difference between the raters. The colored lines show the mean of the paired difference; the black lines show LoAs from −1.96 s to 1.96 s.

The scatter plots in Figure 4 illustrate a high correlation between the two raters regarding mDTI values FA, MD, λ_1,_ and RD for both segmentation methods in dystrophic muscle (ICC ≥ 0.972). Limits of agreement were similar in both techniques in dystrophic muscle, as depicted in the Bland-Altman plots in Figure 5.

**Figure 6:**
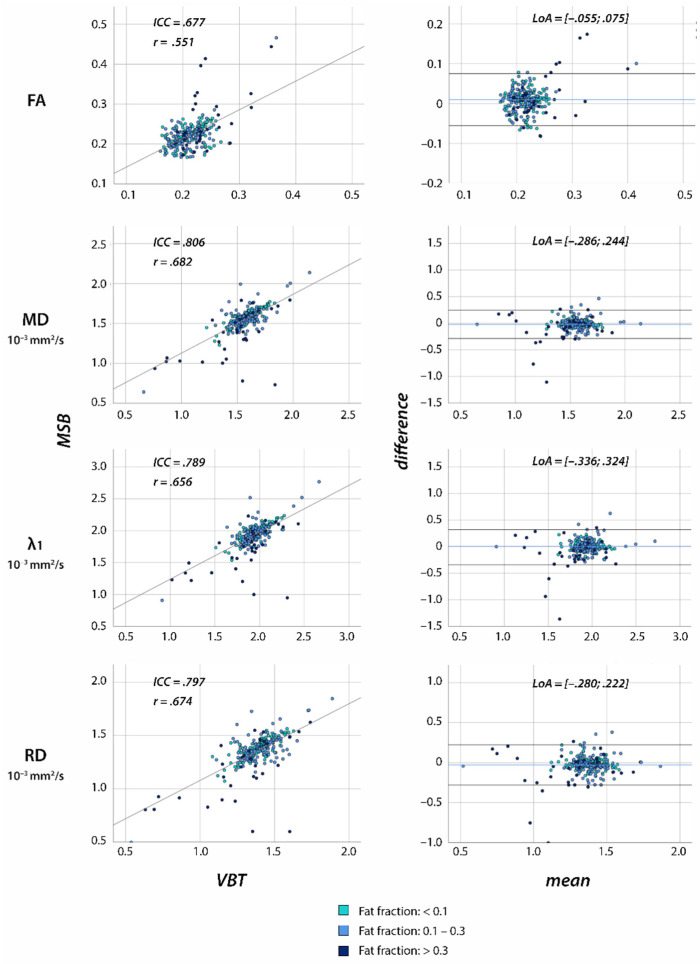
Scatter plots of correlation and Bland-Altman plots between VBT and MSB for one rater in individuals with neuromuscular diseases. Scatter plots include Pearson (r) and intraclass correlation coefficient (ICC). In Bland-Altman plots, the x-value shows the mean of two raters and the y-value the difference between the raters. The colored lines show the mean of the paired difference; the black lines show LoAs from −1.96 s to 1.96 s.

High inter-rater agreement was found for all muscles independent of the extent of fatty infiltration (see Supplementary Table S1). Correlation between methods for the same rater revealed lower reliability between MSB and VBT (ICC ≤ 0.806; see Figure 6). For tract properties, excellent agreement between raters was shown using ICC and Pearson correlation coefficient (see Supplementary Figure S1).

## 4. Discussion

Since diffusion metrics vary between different muscle groups in healthy controls and NMD, muscle segmentation has an important role in the analysis of mDTI data [3]. In NMD, the segmentation process is even more challenging because fatty infiltration, increase in connective tissue, and inflammation complicate differentiation of different muscle groups [12]. In this study, two evaluated segmentation techniques—MSB and VBT—showed excellent inter-rater reliability in healthy and dystrophic muscles and, therefore, a low rater dependency despite a high degree of fatty infiltration. These findings allow us to compare and pool data of NMD patients from different studies and centers, as well as segmentations from different raters. Furthermore, MSB and VBT showed high precision with a comparably low CV for both methods suggesting that diffusion metrics assessed with both methods are suitable for group comparisons.

Between VBT and MSB, no significant difference was observed for λ_1,_ indicating high stability of axial diffusivity in our data and between methods. The significantly higher MD and RD, along with the lower FA values in VBT, suggest a higher sensitivity towards transversal diffusion for VBT in comparison to MSB. Changes in RD with unaffected λ_1_ have been associated with a different myofiber diameter [21]. Thus, the variance between methods may be explained by a higher sensitivity of VBT to myofiber diameters but can also be related to fiber tracking stop criteria (FA range 0.1–0.6).

Additionally, the significant differences between both methods suggest an effect of the segmentation method itself, which likely results from the different weighting of diffusion information. As described previously in MSB, manually segmented muscles volume is superimposed on DWI images, and mDTI data are extracted once for every voxel per volume. In VBT, a tractography algorithm is implemented inside the previously defined muscle volumes, which are superimposed on DWI images. The mean diffusion metrics are then extracted from the mathematically calculated fiber tracts. This leads to a different calculation of mean diffusion metrics since mDTI values of every voxel are summed up with every tract visitation. Due to this spatial weighting and multiple counting of high-quality data voxels, mDTI data are probably less influenced by low SNR regions and partial volume effects in VBT. The different weighting of diffusion information is likely the explanation for the small absolute but significant differences of mean DTI values between both methods. Furthermore, reliability analysis between MSB and VBT revealed a moderate correlation. These findings underline the necessity to harmonize segmentation protocols prior to the comparison of mDTI data.

A recently published study by our group showed higher accuracy of VBT compared to MSB in healthy thigh muscles [6]. In contrast to those findings, similar outcomes for both techniques were found in calf muscles. A potential cause is more artifacts in the thigh muscles due to a bigger field of view and difficulties in coil positioning. Since SNR was high in both healthy and dystrophic calf muscle, the quality of data was sufficient. In this case, the influence of previously mentioned diffusion weighting may be lower than in thigh muscles and, therefore, fewer differences between methods were observed. This effect could be supported by the different architecture of calf muscles as compared to thigh muscles with more straight aligned muscle fibers [22].

VBT has the advantage of providing additional tract properties which can reflect the muscle macrostructure. A field of application of DTI-based tractography is the detection of early signs of muscle tears, which cannot be observed in standard diffusion analysis [23]. In patients with late-onset Pompe disease, changes of diffusion metrics in muscles without fatty infiltration have been described and may reflect structural changes prior to fatty infiltration [24]. A small case series in patients with spinal muscular atrophy treated with Nusinersen did not analyze DTI information quantitatively but showed an increase of fiber tracts over a period of two years while fat infiltration on T1 remained unchanged [25]. Therefore, tract properties may allow an assessment of disease progression in NMD, but currently, there is a lack of clinical studies. In this study, we found excellent inter-rater reliability for tract properties in dystrophic muscles regardless of the extent of fatty infiltration, suggesting high accuracy of VBT and feasibility in clinical studies. Mean tract length in our study showed good agreement with anatomical studies and other studies using advanced tractography techniques like tract-density maps or anatomically constrained tractography [5,7,22]. Comparably higher mean tract length of tibialis anterior muscle may be explained by the homogenous muscle structure resulting in a lower mean angle between fiber tracts.

We would like to address potential limitations in this study. Comparisons of diffusion metrics between healthy control and NMD group were not possible since healthy controls were not age nor height- or weight-matched, and diffusion metrics are known to show age- and BMI-related changes [2,26,27]. Differences in age spectrum and constitution may partly explain the wider variance of DTI indices in the NMD group. Furthermore, we performed full manual muscle segmentation since it is the gold standard for muscle separation, but this segmentation technique is time-consuming and requires experience from the rater. (Semi) Automatic segmentation approaches may offer a significant reduction of segmentation time in the near future [28].

## 5. Conclusions

In conclusion, we have shown that both segmentation techniques can be used in the evaluation of DTI metrics in healthy controls and different NMD with high inter-rater reliability and low coefficients of variation. Significant differences between diffusion metrics and moderate reliability between MSB and VBT suggest an influence of the method itself, which needs further investigation. Since the prevalence of NMD is low, pooling of data is often necessary to achieve sufficient sample groups for clinical studies. Our data underline that the same segmentation protocol must be used to ensure comparability. Tract properties calculated with VBT showed high inter-rater reliability and may offer additional information about muscle macrostructure.

## Abbreviations:

3Dthree-dimensionalCVcoefficient of varianceDWIdiffusion-weighted imagingFAfractional anisotropyFOVfield of viewIBMinclusion body myositisICCintraclass correlation coefficientLGMDlimb-girdle muscular dystrophyMDmean diffusivitymDTImuscle diffusion tensor imagingMSBmanual segmentation-based analysisMTLmean tract lengthNMDneuromuscular diseasesPDproton density-weightedqMRIquantitative magnet resonance imagingRDradial diffusivitySPAIRspectral attenuated inversion recoveryTDtract densityTEecho timeTRrepetition timeTSEturbo spin-echoVBTvolume-based tractographyVolvolume

## Figures and Tables

**Table 1:** Overview of DTI parameters, tract properties and coefficient of variation (CV) for segmentation techniques. MSB = manual segmentation-based analysis, VBT = volume-based tractography, CV = coefficient of variance, NMD = neuromuscular diseases, TD = tract density, MTL = mean tract length, Vol = volume.

		MSB				VBT				Paired *t*-Tests
		Mean		SD	CV	Mean		SD	CV	
Healthy controls *n* = 20	FA	0.22	±	0.03	0.13	0.21	±	0.03	0.12	<0.001
	MD	1.58	±	0.10	0.06	1.60	±	0.12	0.08	0.005
	λ_1_	1.97	±	0.12	0.06	1.98	±	0.14	0.07	0.551
	RD	1.39	±	0.10	0.07	1.42	±	0.12	0.08	<0.001
	TD					25.61	±	2.45	0.10	
	MTL					60.27	±	26.66	0.44	
	Vol					158.9	±	132.0	0.83	
NMD *n* = 18	FA	0.22	±	0.04	0.17	0.21	±	0.03	0.14	<0.001
	MD	1.54	±	0.18	0.11	1.57	±	0.16	0.10	0.025
	λ_1_	1.92	±	0.21	0.11	1.93	±	0.19	0.10	0.621
	RD	1.35	±	0.16	0.12	1.38	±	0.15	0.11	<0.001
	TD					24.85	±	2.81	0.11	
	MTL					53.45	±	26.99	0.50	
	Vol					156.5	±	135.8	0.87	

**Table 2:** Overview of intraclass correlation coefficient and Cronbach’s α for segmentation techniques; ICC = intraclass correlation coefficient, MSB = manual segmentation-based analysis, VBT = volume-based tractography, NMD = neuromuscular diseases.

		MSB			VBT	
		ICC	Cronbach’s α	ICC		Cronbach’s α
Healthy controls *n* = 20	FA	0.991	0.991	0.972		0.972
	MD	0.995	0.995	0.985		0.985
	λ_1_	0.992	0.993	0.980		0.980
	RD	0.993	0.994	0.983		0.983
NMD *n* = 18	FA	0.993	0.993	0.985		0.985
	MD	0.995	0.995	0.989		0.989
	λ_1_	0.994	0.994	0.988		0.988
	RD	0.994	0.994	0.988		0.989

## Data Availability

The data presented in this study are available on request from the corresponding author. The data are not publicly available due to data protection reasons.

## Supplementary Materials

The following are available online at https://www.mdpi.com/article/10.3390/diagnostics11091521/s1, Figure S1: Overview of tract properties of NMD participants extracted with volume-based tractography, Table S1: Overview of intraclass correlation coefficient (ICC) for segmentation techniques with varying fat fractions (FF) in neuromuscular patients.
