# Supplementary material for: High Inter-Rater Reliability of Manual Segmentation and Volume-Based Tractography in Healthy and Dystrophic Human Calf Muscle
Source: Diagnostics (Basel). 2021 Aug 24;11(9):1521. doi: 10.3390/diagnostics11091521 (PMC8466691; doi:10.3390/diagnostics11091521)
Supplement: Supplementary file 1 [file diagnostics-11-01521-s001.zip › Table S1.pdf]

Table S1: *Overview of intraclass correlation coefficient (ICC) for segmentation techniques with varying fat fractions (FF) in neuromuscular patients. MSB = manual segmentation-based analysis, VBT = volume-based tractography.*

		**FF < 0.1**	**FF 0.1 – 0.3**	**FF > 0.3**
**MSB**	FA	0.993	0.993	0.995
	MD	0.991	0.993	0.993
	λ_1	0.984	0.994	0.992
	RD	0.992	0.991	0.994
**VBT**	FA	0.991	0.993	0.991
	MD	0.975	0.983	0.994
	λ_1	0.973	0.979	0.990
	RD	0.979	0.985	0.994
