# Supplementary figures and images for: High Inter-Rater Reliability of Manual Segmentation and Volume-Based Tractography in Healthy and Dystrophic Human Calf Muscle
Source: Diagnostics (Basel). 2021 Aug 24;11(9):1521. doi: 10.3390/diagnostics11091521 (PMC8466691; doi:10.3390/diagnostics11091521)

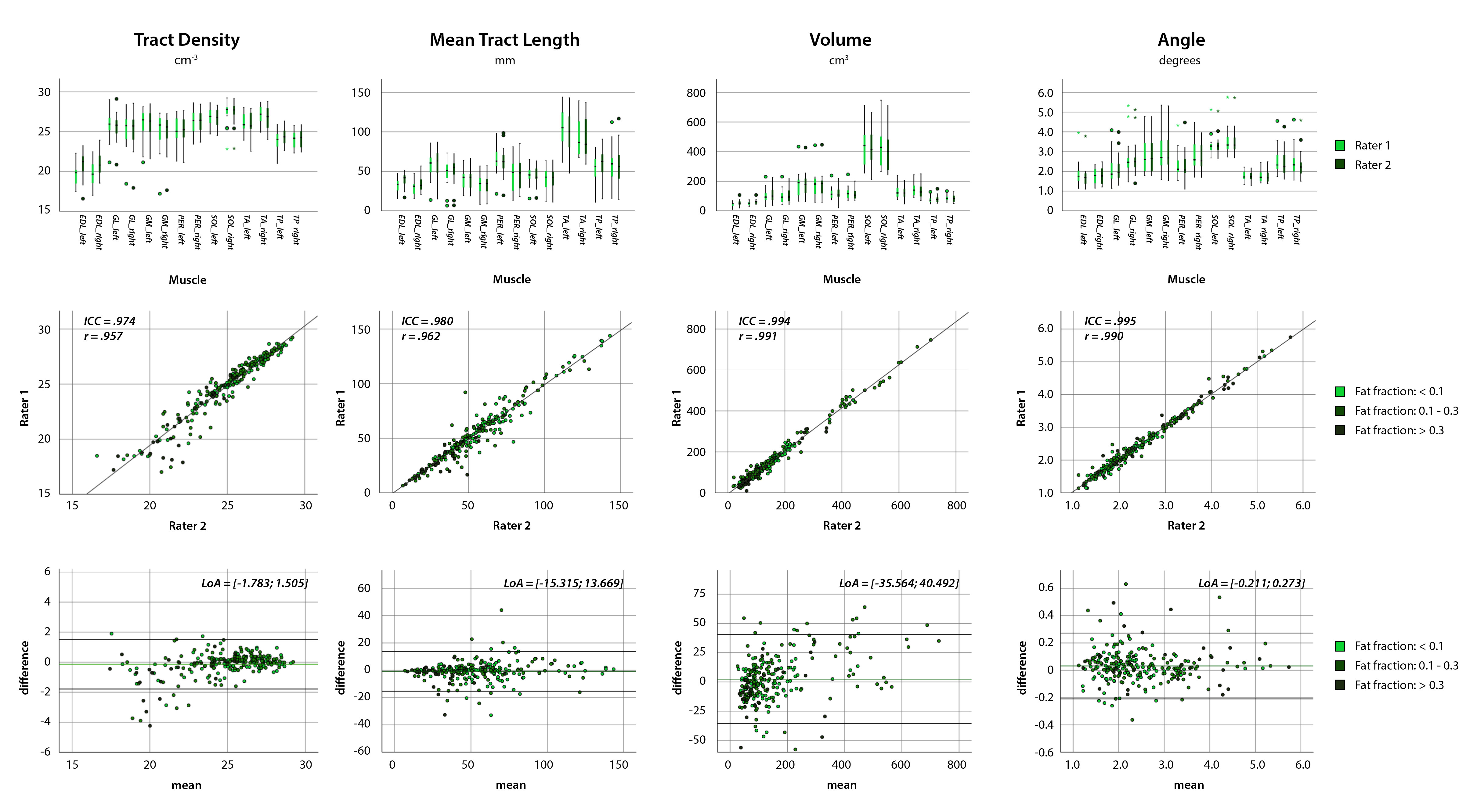

Supplement: Supplementary file 1 [file diagnostics-11-01521-s001.zip › Figure S1.jpg]
